# EHMTI-0320. Orthostatic headache and audiovestibular dysfunction associated with intracranial hypotension

**DOI:** 10.1186/1129-2377-15-S1-D7

**Published:** 2014-09-18

**Authors:** JH Choi, KY Cho, SY Cha, JD Seo, MJ Kim, JS Kim, KD Choi

**Affiliations:** 1 Neurology, Pusan National University Yangsan Hospital, Yangsan, Korea; 2 Neurology, Pusan National University Hospital, Busan, Korea; 3 Neurology, Bonhospital, Busan, Korea; 4 Neurology, Seoul National University Bundang Hospital, Seongnam, Korea

## Introduction and aims

To investigate the patterns and mechanisms of audiovestibular dysfunction in intracranial hypotension.

## Methods

We had consecutively recruited 16 adult patients with intracranial hypotension at the Dizziness Center of Pusan National University Hospital between November 2011 to November 2013. Spontaneous, gaze-evoked, and positional nystagmus were recorded using 3D video-oculography. Most patients had pure tone audiometry, and bithermal caloric tests.

## Results

Out of the 16 patients with intracranial hypotension, five (31%) had neuro-otological symptoms along with the orthostatic headache. One of them presented with recurrent spontaneous vertigo and tinnitus mimicking meniere's disease (MD). Oculographic analysis documented abnormal eye movements in 38%, which include spontaneous downbeat nystagmus with variable positional modulation (n=3, 19%) and positional upbeat nystagmus (n=3, 19%). During the attack of vertigo in the patient with MD-like symptoms, we observed unidirectional horizontal and torsional nystagmus with normal head impulse test. Bithermal caloric tests were normal in all patients who tested. Audiometry showed unilateral or bilateral sensorineural hearing loss in about half of the patients.

## Conclusions

Our study demonstrates that intracranial hypotension can induce higher frequency of audiovestibular dysfunction, which may be attributed to both irritation or dysfunction of the peripheral labyrinth or vestibulocochlear nerve, and brainstem or cerebellar dysfunction due to brain sagging.

No conflict of interest.

